# Astaxanthin protects cognitive function of vascular dementia

**DOI:** 10.1186/s12993-020-00172-8

**Published:** 2020-11-18

**Authors:** Ningwei Zhu, Xiao Liang, Ming Zhang, Xiaolan Yin, Hui Yang, Yajun Zhi, Guizhen Ying, Jialing Zou, Lei Chen, Xiaokun Yao, Hongwei Li

**Affiliations:** 1 Department of Pharmacy, Zhejiang Pharmaceutical College, 888 Yinxian Road, YinZhou District, Ningbo, 315000 Zhejiang China; 2 Department of Neurology, Xiyuan Hospital, China Academy of Chinese Medical Sciences, Beijing, 100091 China; 3 Department of Pharmacy, Ningbo Yinzhou No. 2 Hospital, Ningbo, 315000 Zhejiang China; 4 Department of Gastroenterology, Xiyuan Hospital, China Academy of Chinese Medical Sciences, Beijing, 100091 China

**Keywords:** Vascular dementia, Cognition function, Anti-inflammation, Anti-oxidant, Astaxanthin

## Abstract

**Objective:**

The purpose of this study was to evaluate the effect of astaxanthin (AST) on cognition function, inflammatory response and oxidative stress in vascular dementia (VD) mice.

**Method:**

VD mice model was established by left unilateral common carotid arteries occlusion (LUCCAO). Following LUCCAO, AST was intragastrically administered for 30 days. Object recognition test and morris water maze test were used to evaluate cognitive function. Hematoxylin and eosin staining was performed to observe the hippocampal neuron structure. Enzyme-linked immunosorbent assay kit and bicinchoninic acid kit were respectively adopted to measure IL-1β and IL-4 protein expression and superoxide dismutase (SOD) activity and malondialdehyde (MDA) content in hippocampus and prefrontal cortex.

**Results:**

AST improved the discrimination ability of VD mice. The escape latency and path length of VD mice treated with AST were dramatically reduced. Besides, AST 200 mg/kg enhanced crossing platform time and the number of times crossing the platform quadrant, and alleviated the morphological impairment in VD mice. Moreover, we found that AST inhibited IL-1β expression and MDA content, whereas promoted IL-4 expression and SOD activity in a dose-dependent manner.

**Conclusion:**

AST could improve cognitive impairment and hippocampal neurons in VD mice, which may be related to suppression of inflammatory response and oxidative stress.

## Introduction

Vascular dementia (VD) refers to acquired intelligence disorder syndrome, which is finally caused by the long-term exposure to various risk factors of cerebral vascular diseases such as cerebral ischemia and hypoxic damage [1]. The development of VD negatively impacts patient cognitive function, depression and anxiety, and working memory [2]. In recent years, the incidence of cerebrovascular diseases has been increasing with the acceleration of population aging, as well as the incidence of VD. Although the exact etiopathogenesis of VD remains unclear, numerous reports have shown that inflammatory response and oxidative stress may be the pathogenesis of cognitive dysfunction, resulting in brain structure abnormalities and dysfunction in VD mice [3, 4]. Therefore, it is urgent to identify potential therapeutic drugs for VD.

Astaxanthin (3,3′-dihydroxy-b, b′-carotene-4,4′-dione, AST), a natural carotenoid with high anti-oxidative activity, is widely distributed in algae, crab, shrimp, salmon, and crustaceans [5]. Antioxidant function of AST have been shown in vitro and in vivo, and its anti-inflammatory and anticancer effects have also been reported in several biologic activities [6]. Studies conducted by You et al. and Sila et al. have indicated that AST protects proximal tubular epithelial cells exposed to high glucose, oxidative stress, inflammation, and apoptosis in diabetic nephropathy rats [7, 8]. One recent study has demonstrated that AST exhibits noticeable neuroprotection on ischemia-induced impairment in transient cerebral ischemia mice [9], brain damage induced by ischemia–reperfusion in vivo and H_2_O_2_-induced neurotoxicity in vitro [10]. However, the function of AST on cognitive function of VD mice and its underlying mechanism remain unclear.

Chronic cerebral hypoperfusion (CCH) is a major cause of VD, which can be caused by diseases affecting the cerebrovascular system, including hypertension, diabetes, systemic atherosclerosis and smoking [11]. Ma et al. have adopted mice model of CCH induced by right unilateral common carotid artery occlusion (UCCAO) [12]. This study was designed to analyze the effect of AST on VD mice model induced by left UCCAO (LUCCAO), and to explore whether its effect was involved in inflammatory response and oxidative stress.

## Materials and methods

### Animals

A total of 65 male Institute of Cancer Research (ICR) mice weighting 30–35 g were obtained from Laboratory Animal Center of Zhejiang Experimental Animal Center, and were housed in a light-controlled room (14-h light, 10-h dark cycle) with a consistent temperature (24 ± 2 °C). This study was approved by the Animal Ethics Committee of Zhejiang Pharmaceutical College (2020244). Animal health and behavior were monitored daily.

### Establishment of vascular dementia model

Mice were initially divided into sham group (n = 13) and LUCCAO group (n = 52). These mice were fixed in a supine position after anesthesia with sodium pentobarbital (60 mg/kg). The left common carotid artery was exposed through a midline neck incision, and the artery was ligated with sutures to perform LUCCAO. Mice in sham group underwent same procedure, except for carotid ligation. After operation, mice were placed under a heat lamp to prevent hypothermia until they recovered from general anesthesia completely. After recovery, none of the mice had sign of encephalitis and were maintained in cage in groups.

### AST solution preparation and intragastric administration

Different doses of AST standard solutions (5, 10 and 20 mg/ml, Sigma, USA) were respectively dissolved with 5% sodium carboxymethylcellulose, and then stirred with a vortex mixer 20 min in dark room at room temperature. In the process of administration, mice in LUCCAO group were further subdivided into 4 subgroups and respectively received 5% sodium carboxymethylcellulose, AST 50, 100 or 200 mg/kg with 13 mice per group [13]. Meanwhile, the mice in sham group and model group were injected at 4 p.m. once a day for 30 consecutive days after surgery.

### Object recognition test

To assess non-spatial working memory, a 2-day object recognition test was performed on the 31st day after LUCCAO [14]. The apparatus was constructed using a box (45 × 45 × 40 cm). The objects were made of three different shapes and colors: cubes (green), cylinders (red), and pyramids (blue) of 10 cm height. On the first day of test, the mice were allowed to explore box 5 min without any objects. Next day, two identical objects were presented on two opposite sides of the box, and the mice explored for 10 min in the first trial. On the second day of the test, we put these mice in the box for 3 min, and replace one of the objects presented in the first trial. Directing exploration was considered as the nose at a distance < 1 cm from the object or touching it. To avoid local preference and olfactory stimuli, we randomly changed the role and location of the two objects during the second trial, and cleaned them carefully with 70% alcohol. We manually recorded that the time spent in exploring familiar (F) object and new (N) object. Discrimination index was calculated (N − F/N + F) for intergroup comparison [15].

### Morris water maze test

Morris water maze test was performed to evaluate the spatial acquired function of mice on the 33th day after LUCCAO in accordance with previous researches [16, 17]. A round plastic pool (160 cm in diameter and 55 cm in height) was filled with opaque water (24 ± 2 °C). The pool was divided into 4 quadrants of equal size. A circular platform with a diameter 12 cm was submerged 2 cm below the water in a quadrant. The laboratory was kept dark and quiet during the test.

In probe trial, mice were trained to escape from the water by swimming to the platform. Once the platform was found, mice were allowed to stay on the platform for 10 s. If mice fail to find the platform within 60 s, they would be guided to the platform. During five consecutive days of training, mice were trained 4 times a day for 20 s, with the platform in the same position relative to the distal cues in the room. In each trial, mice were placed in the water at different start locations (east, south, west and north). After 24 h of training, the hidden platform trial was performed to assess consolidation and retrieval of memory, mice swam without the platform. All mice launched at the same place and time, and the number of crossing target quadrant and the original platform area were observed. Data were collected by an online video tracking device (RD1101-MWM-G, Moblie Datum Inc, Shanghai, China).

### Collection of mice hippocampal tissues

At the end of above experiments, the mice were anesthetized via sodium pentobarbital (60 mg/kg) until muscular flaccidity. We verified mice death by respiratory and cardiac arrest. Brains were rapidly removed from the cerebrum of the mice, and the best efforts were made to minimize animal suffering. One part of brains was snap frozen using liquid nitrogen, and then stored at 80 °C for enzyme-linked immunosorbent assay (ELISA) and Bicinchoninic acid analysis. The other part of brains was fixed in 4% paraformaldehyde at 4 °C for 24 h, followed by dehydration and embedding with paraffin for analysis using histology techniques.

### Hematoxylin and eosin staining

To observe the hippocampal neuron structure of mice, H&E staining was adopted. Paraffin-embedded brain tissue was cut into 5 µm sections from optic chiasma to cerebral transverse fissure on a microtome, following deparaffinization in xylene and rehydration with graded ethanol. The sections were stained using H&E staining kit (Beyotime, Shanghai, China). Results were observed under the optical microscope (40 × 10) (ERc5S, Carl Zeiss Company).

### Enzyme-linked immunosorbent assay

Protein levels of interleukin-1β (IL-1β) and interleukin-4 (IL-4) in the hippocampus and prefrontal cortex were measured to investigate the regional response of pro-inflammatory and anti-inflammatory cytokine of mice by ELISA kit (Beyotime, Shanghai, China). Briefly, serial dilutions of protein standards and samples of mice were added to ELISA plates, followed by biotinylated anti-IL-1β and IL-4 antibody addition. Then, the prepared solution of avidin-horseradish peroxidase conjugate complex was added, and the unbound conjugates were washed away with phosphate buffered saline. The reaction was stopped by adding stopping solution, and absorbance was read at 450 nm.

### Detected of superoxide dismutase (SOD) and malondialdehyde (MDA)

To further determine whether AST influences anti-oxidation, we analyzed SOD activity and MDA content in the hippocampus and prefrontal cortex of each group. All hippocampus tissue were obtained after normal perfusion (0.9% NaCl). A 10 mg hippocampus tissue was weighed and added to 100 ml SOD sample preparation fluid. The mixture was homogenized at 4 °C or ice bath. Then supernatants were obtained and used as a sample to be tested after centrifugation at 12,000*g* for 3–5 min at 4 °C. Bicinchoninic acid kit used to determine the protein concentration of each sample, and SOD and MDA tests were performed according to kits steps (Beyotime, Shanghai, China) [18].

Calculation of total SOD activity in samples: SOD enzyme activity units = percent inhibition/{1 − (A blank control 1 − A sample)/(A blank control 1 − A blank control 2) × 100%} units. Then convert the SOD activity units into U/g or U/mg protein [18, 19].

Determination of MDA content in samples: the initial concentration of MDA in the sample solution was determined by the protein concentration per unit weight to the tissue weight, such as μmol/mg of protein or tissue.

### Statistical analysis

All data were statistically analyzed with SPSS 15.0 (International Business Machines Corporation, IBM, USA). For two-way analysis of variance (ANOVA), the procedure (sham) and the treatment (AST 50, 100, 200 mg/kg) were taken as between-group factors. One-way ANOVA with Tukey’s post hoc test was used for multiple comparisons to determine whether the means differed significantly between two groups. A value of P ≤ 0.05 was considered statistically significant, results were presented as mean ± SEM.

## Result

### Weight of mice

**Fig. 1:**
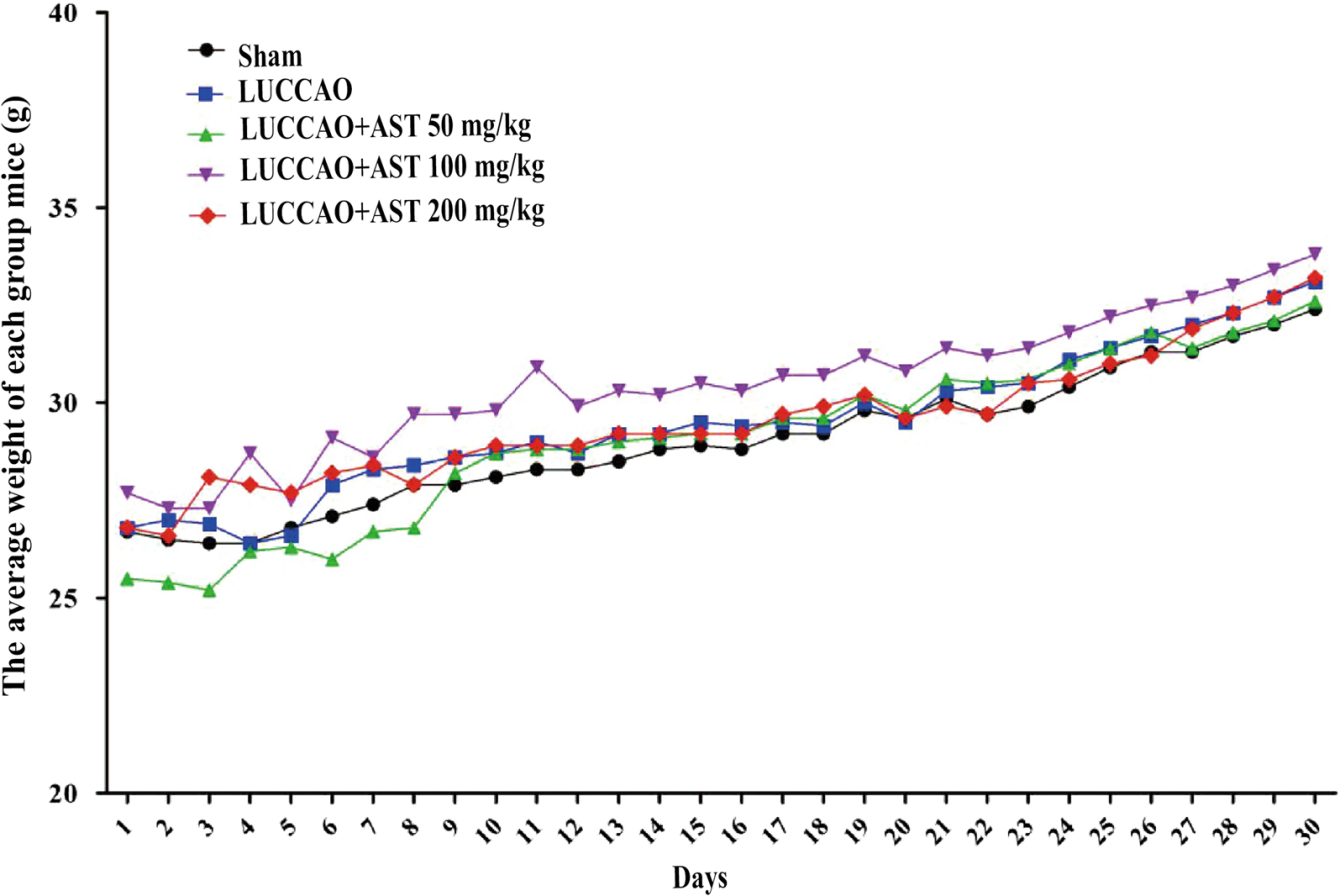
The weight of each group mice in the 30 days, with 13 mice per group

During the period of dministration, the average body weight of each group increased steadily for 30 days, except for slight fluctuations in the first ten days. But there was no significant difference between each group (P > 0.05) (Fig. 1), indicating that AST did not affect the body weight of mice.

### AST improves non-spatial cognitive function in VD mice

**Fig. 2:**
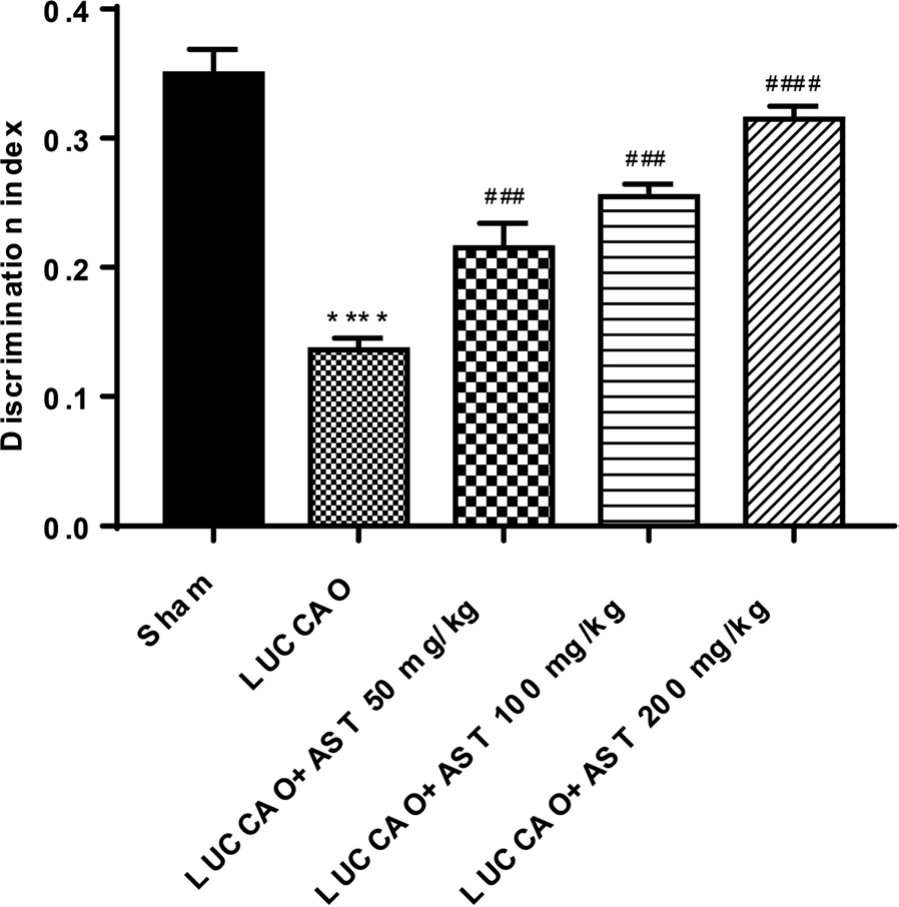
The discrimination index of different groups, with 13 mice per group. (****P < 0.0001: compared with sham group; ^###^P < 0.001, ^####^P < 0.0001: compared with LUCCAO group)

After LUCCAO, mice exhibited a significant reduction in discrimination ability when compared with mice in sham group (P < 0.0001) (Fig. 2). Whereas, the discrimination indices of mice treated with AST (50, 100 and 200 mg/kg) were dramatically higher than that in the model group (P < 0.001, P < 0.001 and P < 0.0001, respectively). Taken together, these data indicated that AST could improve the non-spatial cognitive function in mice with VD.

### AST ameliorates spatial cognition impairment in VD mice

**Fig. 3:**
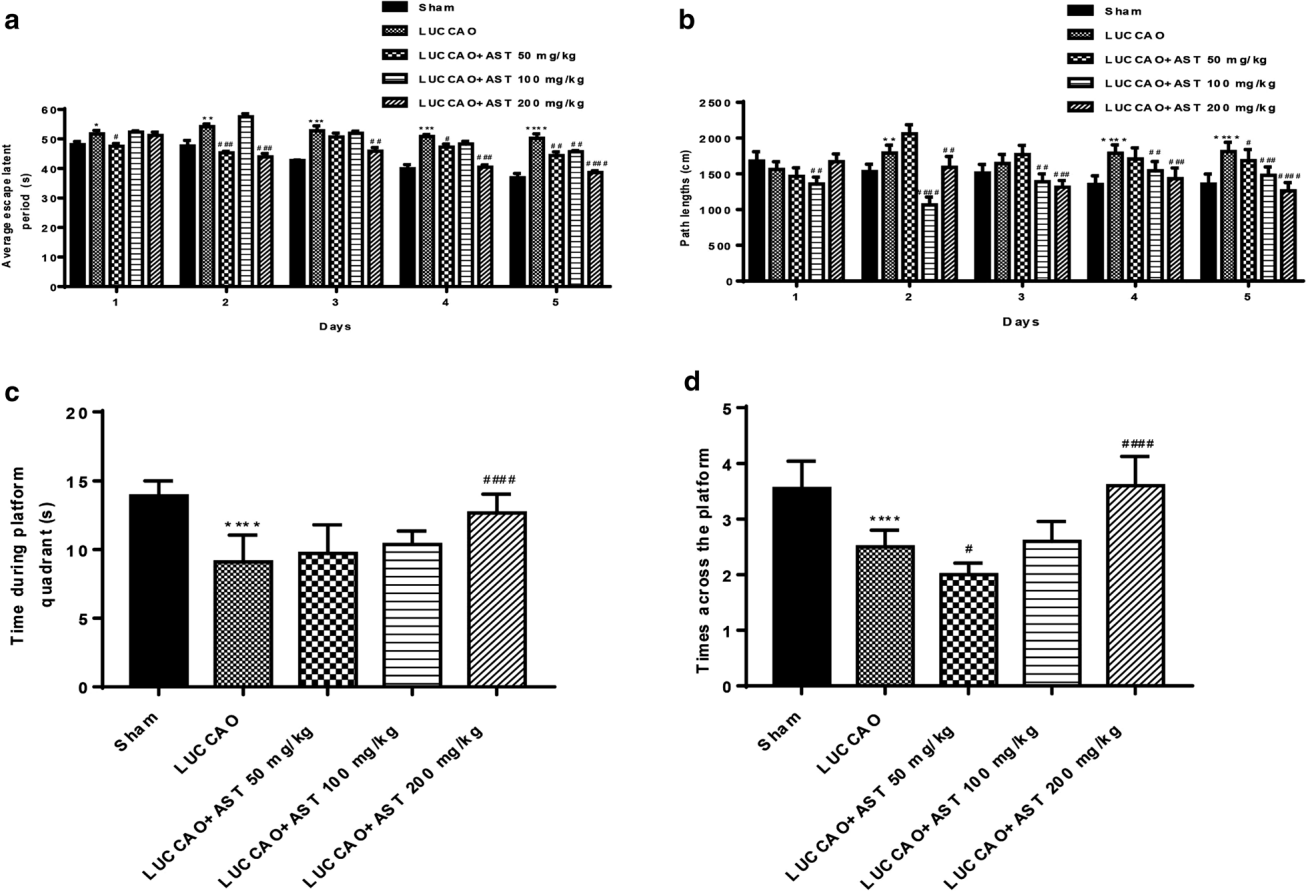
The results of morris water maze test, with 13 mice per group. **a** The average escape latency period of every group in the five days. **b** The path length of each group in the five days. **c** Time for mice crossing platform quadrant. **d** The number of times of mice cross the platform (*P < 0.05, **P < 0.01, ***P < 0.001, ****P < 0.0001: compared with sham group; ^#^P < 0.05, ^##^P < 0.01, ^###^P < 0.001, ^####^P < 0.0001: compared with LUCCAO group)

As shown in Fig. 3a, b, LUCCAO-treated mice had longer escape latency and path length when compared with sham group, which were markedly shortened after administration of AST 200 mg/kg. LUCCAO induced a decrease in crossing platform time and the number of times crossing the platform quadrant, which were also reversed by treatment of AST 200 mg/kg [F (4, 60) = 22.28, P < 0.0001; F (4, 60) = 40.97, P < 0.0001] (Fig. 3c, d). These findings indicated that AST might ameliorate the impairment of spatial acquired function to a certain extent.

### Effect of AST on hippocampus neuron in VD mice

**Fig. 4:**
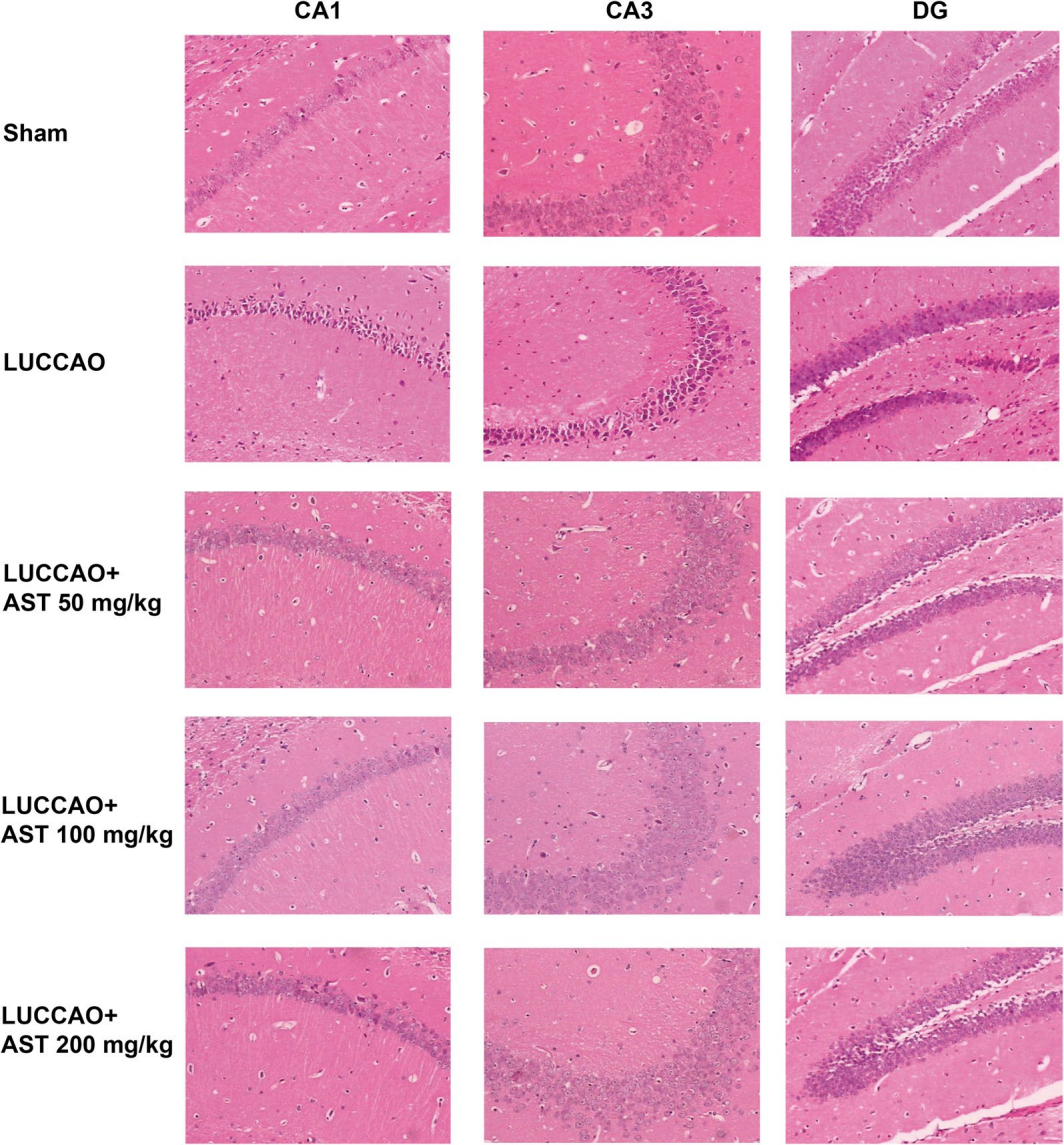
The hematoxylin and eosin staining results of each group, with 13 mice per group. Bar: 100 um

Results showed that the hippocampal neuron structure in LUCCAO-treated mice was significantly damaged, and their nuclei was lost (Fig. 4). In particular, the neurons in the hippocampal CA1 and CA3 area of mice in model group contracted seriously and the adjacent gap were enlarged, with disordered and hyperchromatic arrangement of neurons. After AST treatment, the damage of neuron was alleviated, and the neurons in CA1 and CA3 areas were remarkably improved, with orderly arrangement and large and clear nucleus. These findings indicated that AST could alleviate the morphological impairment caused by VD in mice.

### AST regulates IL-1β and IL-4 expression in the hippocampus and prefrontal cortex of VD mice

**Fig. 5:**
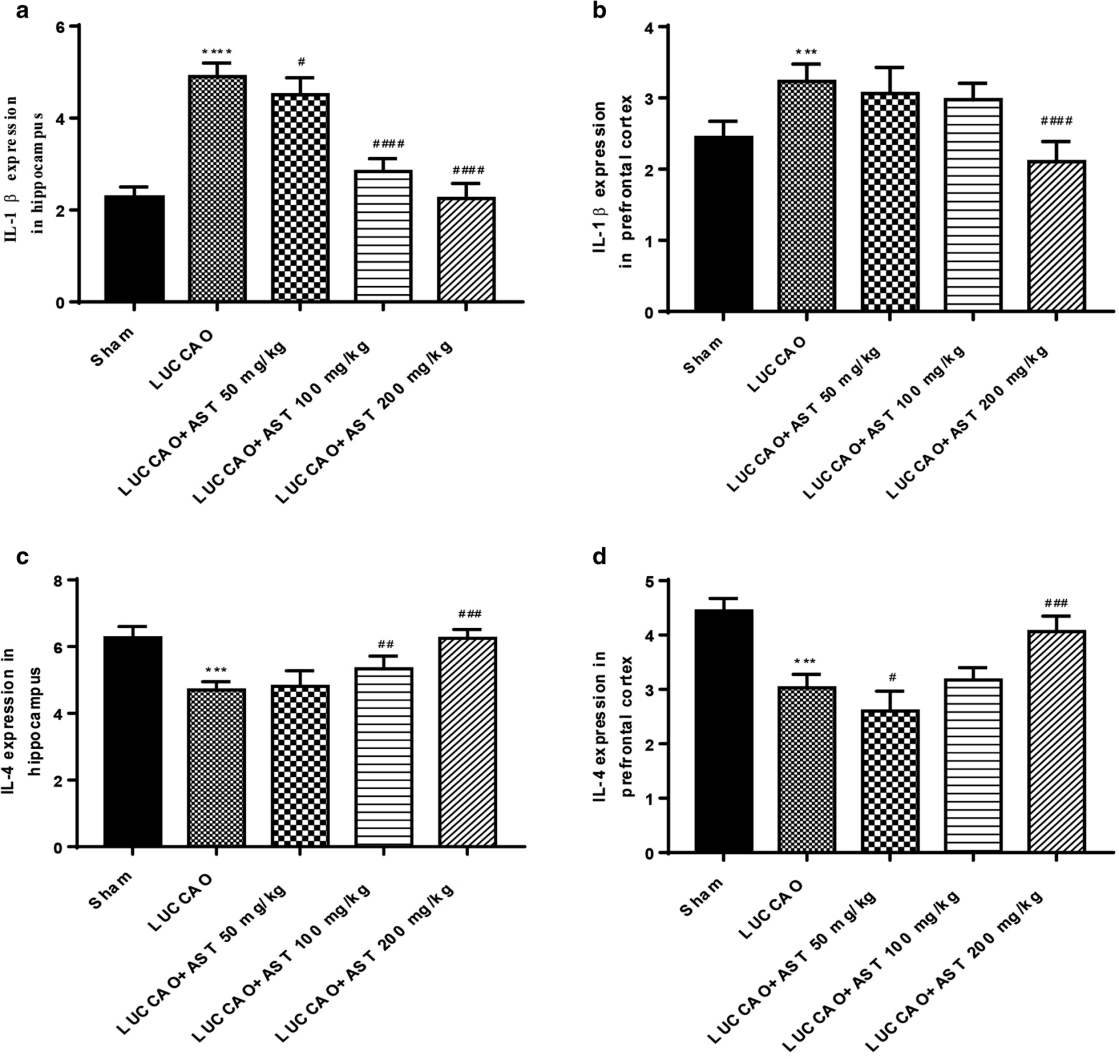
The expression of IL-1β and IL-4 in the hippocampus and prefrontal cortex of each group mice, with 13 mice per group. **a** The expression of IL-1β in the hippocampus. **b** The expression of IL-1β in the prefrontal cortex. **c** The expression of IL-4 in the hippocampus. **d** The expression of IL-4 in the prefrontal cortex (***P < 0.001, ****P < 0.0001: compared with sham group; ^#^P < 0.05, ^###^P < 0.001, ^####^P < 0.0001: compared with LUCCAO group)

IL-1β level in the LUCCAO-treated mice was significantly elevated compared to that in sham group in the hippocampus and cerebral cortex. However, AST dose-dependently abated IL-1β expression in the hippocampus (P < 0.05), and only AST 200 mg/kg lowered IL-1β expression in cerebral cortex (P < 0.0001) (Fig. 5a, b). Besides, AST elevated the expression of IL-4 in the hippocampus and prefrontal cortex in a dose-dependent manner when compared with that in LUCCAO group, and reached the highest at AST 200 mg/kg (P < 0.0001) (Fig. 5c, d) According to the above results, it was implied that AST could regulate the expression of inflammatory cytokines in VD mice.

### AST suppresses oxidative stress in the hippocampus and prefrontal cortex of VD mice

**Fig. 6:**
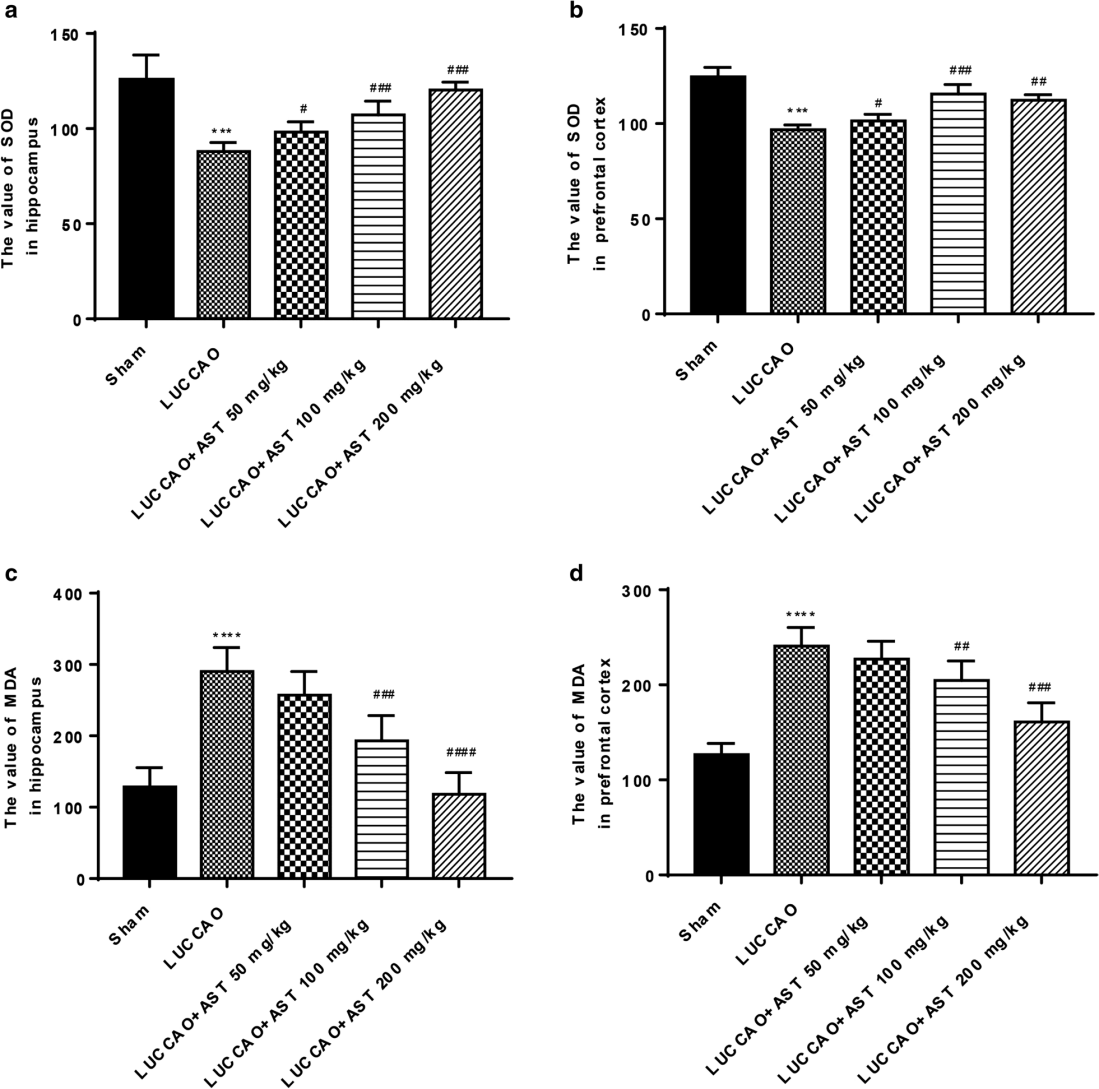
SOD activity and MDA content in the hippocampus and prefrontal cortex of each group, with 13 mice per group. **a** The value of SOD activity in the hippocampus. **b** The value of SOD activity in the prefrontal cortex. **c** The level of MDA in the hippocampus. **d** The level of MDA in the prefrontal cortex (***P < 0.001, ****P < 0.0001: compared with sham group; ^#^P < 0.05, ^##^P < 0.01, ^###^P < 0.001, ^####^P < 0.0001: compared with LUCCAO group)

From Fig. 6a, b, we found that SOD activity was noticeably decreased in the hippocampus and prefrontal cortex of LUCCAO group (P < 0.001). However, mice treated with AST (50, 100, and 200 mg/kg) had higher SOD activity in the hippocampus and prefrontal cortex, and the maximal effect was achieved when AST dose was 200 mg/kg (P < 0.05). Compared with sham group, LUCCAO group showed a significant increase of MDA content in the hippocampus and prefrontal cortex (both P < 0.001), which was diminished after AST treatment (100 and 200 mg/kg) in a dose dependent manner (P < 0.01) (Fig. 6c, d). These findings clarified that AST could inhibit the level of oxidative stress.

## Discussion

The most significant finding of this study was that AST ameliorated the cognitive function and hippocampal neuron in VD mice, which may be associated with the inhibition of inflammatory response and oxidative stress, revealing that the potential value of AST in the treatment of VD.

VD is recognized as the second most common type of dementia following Alzheimer’s disease, which is caused by a reduction in blood supply due to blockage in the vascular system, leading to a gradual decline in memory and cognitive function [20]. Currently, many VD animal models have been established, among which 2VO rat model is a classic VD model of whole cerebral ischemia. Numerous studies have shown that permanent hypoxia and hypoperfusion of brain induced by bilateral common carotid arteries (2VO) in rat model can lead to VD and oxidative stress of brain neurons, and ultimately cholinergic dysfunction and decreased learning and memory ability [21, 22]. However, 2VO is only limited to rats and is not suitable for mice, because rats have a complete circle of Willis. Mice lacking complete circle of Willis will suffer from severe ischemia and even death if perform 2VO [23]. Recently, UCCAO model is adopted for VD mice model, which is modified from 2VO model [24]. After 28 days of permanently ligated of a common carotid artery, cerebral perfusion in the ipsilateral hemisphere of mice declined by 35–55% [25]. In this study, we established VD mice model induced by LUCCAO.

Due to the complex pathogenesis of VD-related cognitive dysfunction, there is still no effective treatment. Therefore, it is necessary to find a specific and effective therapeutic drug. Recent studies have shown that AST has the effect of anti-inflammation, anti-apoptosis, anti-oxidation, anti-aging, anti-tumor, and boosting immunity [26, 27]. In the central nervous system (CNS), AST is regarded as a potential neuroprotective drug because of its powerful antioxidant property [28]. In this study, AST alleviated cognitive dysfunction and hippocampal structural damage to a certain extent, indicating that AST had therapeutic effects on VD cognitive dysfunction. In addition, many previous studies have shown that AST can be used as a protective agent in ischemia model owing to its antioxidant potential [29, 30], which is consistent with this study.

It has been demonstrated that inflammatory response and oxidative stress are involved in VD-related cognitive impairment [31, 32]. Overexpression of inflammatory factors can lead to neuron damage in the hippocampus, thus affecting the cognitive function of mice [33]. Miao et al. have confirmed that the levels of interleukin-6 (IL-6), tumor necrosis factor-a, and COX-2 are increased in the brain tissue of type 2 diabetes mellitus rats with cognitive dysfunction [34]. Research conducted by Paloma Bermejo et al. has found that inflammatory response may be an early factor in the development of Alzheimer’s disease [35]. The above studies demonstrate that inflammatory cytokines play a role in the pathogenesis of cognitive dysfunction. In the present study, we found that pro-inflammatory cytokine IL-1β level was increased, while anti-inflammatory IL-4 level was decreased in VD mice, which was consistent with the view that inflammatory cytokines were associated with the pathogenesis of VD-related cognitive dysfunction. However, AST treatment remarkably lowered the IL-1β expression and enhanced IL-4 expression in the hippocampus and prefrontal cortex, indicating that the alleviation of AST in cognitive impairment may correlated with suppression of inflammatory response.

Under normal and non-stress conditions, oxidation and antioxidants levels are relatively balanced, but are out of balance under harmful external stress, thereby leading to the mass production of reactive oxygen species and the relative shortage of antioxidants [36]. Subsequently, a large amount of reactive oxygen species in the organism results in the formation of MDA. MDA is a vital indicator of lipid peroxidation and reflects the severity of oxidative stress injury [37]. In addition, as a free radical scavenger, SOD can protect brain tissue from oxidative stress damage [38]. Mamun et al. have also confirmed that ATX can inhibit neuronal oxidative stress caused by aluminum chloride and ameliorate spatial memory impairment in mice [39]. In this current study, MDA production was elevated and SOD activity was diminished in the hippocampus and prefrontal cortex after LUCCAO, which were significantly reversed by AST treatment. Therefore, we hypothesized that ATX could play a neuroprotective role in cognitive dysfunction of VD mice through its antioxidant effect. Oxidative stress caused by VD can damage the structure of neurons. The results of this study presented damaged hippocampal neuron structure and less nucleus in VD mice, which were alleviated by ATX, indicating that ATX might play a neuroprotective role through anti-oxidative stress and reduced the hippocampal injury of VD mice.

There are some limitations of the study. AST may reduce the damage of neurons in hippocampus induced by VD through oxidative stress, but the apoptosis of neurons was not detected. Besides, we did not detect VD markers such as S100B, C-reactive protein and IL-6. Further studies on these issues were required to carried out.

In summary, this study proves the existence of behavioral impairment in VD mice. Additionally, AST has a significant protective effect on VD mice induced by LUCCAO, which can reduce oxidative stress and enhance the inflammatory levels in a dose-dependent manner. Furthermore, AST 200 mg/kg shows the best neuroprotection property among the three dosages.

## Data Availability

The datasets used and/or analyzed during the current study are available from the corresponding author on reasonable request.

## Abbreviations

VD: Vascular dementia
AST: Astaxanthin
CCH: Chronic cerebral hypoperfusion
LUCCAO: Left unilateral common carotid artery occlusion
ICR: Institute of Cancer Research
ELISA: Enzyme-linked immunosorbent assay
SOD: Superoxide dismutase
MDA: Malondialdehyde
IL-1β: Interleukin-1β
IL-4: Interleukin-4
IL-6: Interleukin 6
2VO: Bilateral common carotid occlusion
